# Development of a novel PCV2 and PCV3 vaccine using virus-like vesicles incorporating Venezuelan equine encephalomyelitis virus-containing vesicular stomatitis virus glycoprotein

**DOI:** 10.3389/fvets.2024.1359421

**Published:** 2024-05-22

**Authors:** Ying Wang, Min Su, Yongshuang Huang, Jianle Ren, Sheng Niu, Yujun Zhao, Fang Yan, Yi Yan, Wen-xia Tian

**Affiliations:** College of Veterinary Medicine, Shanxi Agricultural University, Jinzhong, China

**Keywords:** VEEV, VSVG, virus-like vesicles, PCV2, PCV3

## Abstract

Porcine circovirus disease (PCV) causes substantial economic losses in the pig industry, primarily from porcine circovirus type 2 (PCV2) and porcine circovirus type 3 (PCV3). Novel vaccines are necessary to prevent and control PCV infections. PCV coat proteins are crucial for eliciting immunogenic proteins that induce the production of antibodies and immune responses. A vaccine platform utilizing Semliki Forest virus RNA replicons expressing vesicular stomatitis virus glycoprotein (VSV-G), was recently developed. This platform generates virus-like vesicles (VLVs) containing VSV-G exclusively, excluding other viral structural proteins. In our study, we developed a novel virus-like vesicle vaccine by constructing recombinant virus-like vesicles (rVLVs) that also express EGFP. These rVLVs were created using the RNA replicon of Venezuelan equine encephalomyelitis (VEEV) and New Jersey serotype VSV-G. The rVLVs underwent characterization and safety evaluation *in vitro*. Subsequently, rVLVs expressing PCV2d-Cap and PCV3-Cap proteins were constructed. Immunization of C57 mice with these rVLVs led to a significant increase in anti-porcine circovirus type 2 and type 3 capsid protein antibodies in mouse serum. Additionally, a cellular immune response was induced, as evidenced by high production of IFN-γ and IL-4 cytokines. Overall, this study demonstrates the feasibility of developing a novel porcine circovirus disease vaccine based on rVLVs.

## 1 Introduction

Circoviruses, classified within the family Circoviridae and genus Circovirus (1), represents the smallest autonomously replicating, envelopeless, single-stranded, circular DNA viruses (2). In pigs, four types of circoviruses have been identified: porcine circovirus type 1 (PCV1), porcine circovirus type 2 (PCV2), porcine circovirus type 3 (PCV3), and porcine circovirus type 4 (PCV4) (3–5). PCV2 is associated with post-weaning multisystemic wasting syndrome (PMWS) (6). Since its discovery in 1998, PCV2 has undergone significant evolution, resulting in eight subtypes, from PCV2a to PCV2h. The predominant genotypes historically include PCV2a, PCV2b, and PCV2d. PCV2 has undergone two genotype shifts during its evolution. In 2000, PCV2b notably replaced PCV2a as the predominant genotype (7). Subsequently, after 2010, there was a decline in PCV2b prevalence, coinciding with a rise in PCV2d genotype, which eventually became the predominant genotype (8). PCV3, an emerging porcine circovirus, was first detected in domestic pigs in the United States in 2016. It is associated with porcine dermatitis, nephropathy syndrome (PDNS), reproductive disorders, and cardiac and multisystemic inflammation (4, 9). PCV3, although recently discovered, has been spreading among pigs for many years. Retrospective studies have revealed its presence in pigs before the onset of significant clinical issues (7). Similar to PCV2, changes in porcine susceptibility, pathogenesis, and other factors associated with PCV2 infection may exacerbate PCV2 infection. PCV3 is highly transmissible across species, evolves rapidly, and may cause greater economic harm than PCV2 (10). Controlling novel PCVs is crucial to limit the future transmission of diverse genotypes.

The genome size of porcine circoviruses varies from 1,758 to 2,001 nt (11). It contains two major ORFs, ORF1 and ORF2, encoding viral replication-associated proteins (Rep and Rep’) and the sole capsid protein (Cap), respectively (12). The capsid is a primary source of antigenic epitopes and a common target for vaccine development, including PCV2-inactivated vaccines (13). Subunit vaccines containing baculovirus-expressing PCV2 Cap proteins and inactivated chimeric PCV1-2 vaccines against this pathogen are available (14). PCV2 infection rates continue to rise annually, challenging vaccination programs’ effectiveness in halting PCV2 transmission (15). Presently, there is no effective vaccine or drug for treating or preventing PCV2 infections, and no vaccine exists for PCV3 control. The 36% homology between PCV3 and PCV2 ORF2 genes (16), result in a lack of cross-reactivity, meaning PCV2 vaccines do not protect against PCV3. Consequently, novel vaccines are needed to protect against both PCV2 and PCV3.

Alphavirus-based vectors are engineered for high protein expression in mammalian cells. The most commonly used delivery vectors are based on three types of alphaviruses: Semliki Forest virus (SFV), Sindbis virus (SINV), and Venezuelan equine encephalitis virus (VEEV) (17). Early studies using alphavirus vectors discovered virus-like vesicles (VLV) as specialized vectors that can express exogenous proteins without strict limitations on the size of the inserted fragment (18). VLV is an infectious vesicle composed of an RNA replicon from SFV and a glycoprotein from vesicular stomatitis virus (VSV-G) (19). In initial studies, the VLV genome underwent modifications by incorporating an additional subgenic promoter at the 3′ end of the gene encoding VSV-G to facilitate the expression of extra exogenous proteins. However, this approach posed a risk of potential loss of the exogenous genes from the vector after transmission (18). Subsequently, the VLV genome has been genetically engineered to incorporate a ribosomal T2A skipping site downstream of the exogenous gene and upstream of the VSV-G gene. Which enables the stable expression of exogenous proteins during successive passages (20). Building on relevant, Reynolds et al. leveraged studies involving the 2A peptide to develop vaccines expressing the Hepatitis B virus (HBV) middle surface envelope glycoprotein (MHBs) and hepatitis B core antigen (HBcAg) using VLV as a vector (21). Yarovinsky et al. (22) demonstrated the co-expression of MHBs, HBcAg, and polymerase (mutPol) proteins of HBV using the 3 × T2A sequence. The feasibility capability of expressing additional exogenous proteins through self-cleavage of 2A peptide sequences using virus-like vesicles as vectors has been demonstrated, and various studies have shown that VLVs have the potential to express additional antigenic proteins.

In this study, we engineered VLVs to express exogenous genes, creating recombinant virus-like vesicles (rVLVs) that express EGFP using the RNA replicon of VEEV and New Jersey serotype VSV-G. This confirmed the capability of VEE virus-like vesicles to express exogenous proteins. Subsequently, we constructed rVLVs-PCV2d Cap and rVLVs-PCV3 Cap and confirmed their genetic stability in BHK-21 cells. Finally, we conducted animal experiments to assess the safety and immunogenicity of these rVLVs. These findings have implications for the development and implementation of novel vaccines targeting PCV2 and PCV3.

## 2 Methods

### 2.1 Ethics statement

All experiments were approved by the Institutional Animal Care and Use Committee of Experimental Animal Management at Shanxi Agricultural University, China (Approval No. SXAU-EAW-2023M.BY.006001136).

### 2.2 Cells and recombinant viruses

For cell culture, Hamster BHK-21 (BHK) epithelial cells were cultured in Dulbecco’s modified Eagle’s medium (DMEM; Cytiva) supplemented with 10% fetal bovine serum (FBS; Excell Bio, Australia), 100 U/mL of penicillin, and 100 mg/mL of streptomycin. The porcine circovirus type 2 inactivated vaccine (WH strain obtained from Keqin Biology Co., Ltd., Wuhan, China) was used in this study. The EGFP sequence was amplified using plasmid PEGFP-N1 as a template. The PCV2d Cap (GenBank accession #MK604495.1) and PCV3 Cap (GenBank accession #MN431641.1) genes were synthesized and constructed with HA tags. PCR amplification of the blistering and New Jersey serotype glycoprotein genes. Ribosomal T2A skipping sites (23) were introduced between EGFP and VSV-G, PCV2 Cap and VSV-G, and PCV3 Cap and VSV-G. The primers used in the experiments are listed in Supplementary Table S1. They were cloned into the α-viral VEEV replicon expression vector at its characteristic NdeI and MluI sites. The obtained plasmids, including VEEV—EGFPT2AG, VEEV-PCV2d, CapT2AG, and VEEV-PCV3 CapT2AG were verified by DNA sequencing. Subsequently, the plasmids were linearized with MuI and transcribed *in vitro* using the mMESSENGER mMACHINE T7 Kit (cat. No. AM1344, Ambion, Fisher Scientific Inc.) per the manufacturer’s instructions. RNA transcripts (1 μg) were then transfected into BHK-21 cells using Lipofectamine2000 (cat. no. 11668-019, Ambion, Fisher Scientific Inc.) to obtain rVLVs-EGFP, rVLVs-PCV2d Cap, and rVLVs-PCV3 Cap, respectively, following the instruction’s manual. After observing cellular changes, such as rounding or detachment, the supernatant was collected by centrifugation and stored at 80°C for subsequent experiments.

### 2.3 Virus titration and growth curves

The titers of rVLVs-EGFP, rVLVs-PCV2d Cap, and rVLVs-PCV3 Cap were determined in BHK-21 cells using a standard empty-spot assay. BHK-21 cells were seeded in 12-well plates and infected with 10-fold serial dilutions of the virus. After 1 h after incubation at 37°C, the medium was aspirated, and the cells were washed twice with PBS. Subsequently, 1 mL of DMEM containing 2% FBS and 1% methylcellulose was added to each well. The plates were then incubated for 3 days at 37°C under 5% CO2, followed by fixation in 10% (v/v) formaldehyde for 30 min and staining with 0.05% (w/v) crystal violet to observe empty spots. Growth curves of rVLVs-EGFP, rVLVs-PCV2 Cap, and rVLVs-PCV3 Cap in BHK-21 cells were performed in six-well plates at a multiplicity of infection (MOI) of 0.01. Cell supernatants were collected every 12 h after infection, and the viral titer was quantified using an empty spot assay on BHK-21 cells. VLV growth curves were established based on viral titers at different time points after infection.

### 2.4 Indirect immunofluorescence assay

BHK-21 cells were seeded in 24-well cell culture plates and infected with rVLVs-PCV2 Cap and rVLVs-PCV3 Cap viruses at an MOI of 0.01. After 24 h, the supernatant was discarded, and cells were fixed in 4% cold paraformaldehyde for 30 min at room temperature. The cells were then washed three times with PBS and permeabilized with 0.05% Triton X—100 for 10 min at room temperature followed by three additional washes with PBS. Next, the cells were blocked with 1% BSA, and murine anti-HA-tag mAb was added. After overnight incubation at 4°C, cells were washed 3 times with PBS and then incubated with goat anti-mouse IgG antibodies conjugated with FITC (Bost, Wuhan, China) at 37°C for 1 h. Finally, the cells were then washed with PBS, fixed with 90% glycerol, and observed under a fluorescence microscope (Leica, Germany).

### 2.5 Western blotting analysis

BHK-21 cells were infected with rVLVs-PCV2d Cap and rVLVs-PCV3 Cap at an MOI of 0.1. At 72 hpi, RIPA cell lysate (Beyotime, China) was added and lysed at 4°C for 1 h. Cell samples were separated by 12% SDS-PAGE and electrotransferred onto 0.2 mm polyvinylidene fluoride (PVDF) membranes, which were blocked with 5% skim milk in TBST for 2 h at room temperature. The membranes were incubated with a primary antibody of the anti-HA-tag mAb with 1:2,500 dilution or with the sera of infected pigs with PCV2 or PCV3 at 1:100 dilution at 4°C overnight. Purified PCV2 and PCV3 Cap were separated using 12% SDS-PAGE and the same steps described above for membrane transfer. The membranes were incubated with the sera of infected mice with rVLVs-PCV2 Cap, rVLVs-PCV3 Cap, and DMEM at a 1:100 dilution at 4°C overnight. After washing three times with TBST, the membranes were incubated with horseradish peroxidase (HRP)-labeled secondary antibodies at room temperature for 1 h. After washing the membrane three times with TBST, the protein bands were visualized using an ultrasensitive ECL chemiluminescent reagent (Wanleibio).

### 2.6 Purification of VLV

Five vials of T175 BHK-21 cells were infected with rVLVs-EGFP, rVLVs-PCV2d Cap, and rVLVs-PCV3 Cap at an MOI of 0.01 and incubated for 48 h at 37°C. The supernatants of infected cells were recovered by centrifugation at 5,000 g for 30 min at 4°C. The viral solution, after filtration through a 0.22 μm filter (Merck Millipore), was added to a final concentration of 8% polyethylene glycol-8000 (PEG8000) and precipitated at 4°C overnight. After precipitation, the supernatant was discarded by centrifugation at 14,000 g for 1.5 h at 4°C and the precipitate was resuspended in 1 mL of PBS. The precipitate was then layered onto appropriate amounts of 20%, 30%, 40%, 50%, and 60% sucrose, followed by centrifugation at 4°C, 38,000 rpm for 2 h in an ultrahigh-speed centrifuge (Beckman Coulter, America). The precipitate was resuspended in 100 μL of PBS and stored at −80°C for backup.

### 2.7 Transmission electron microscopy

The purified rVLVs-EGFP, rVLVs-PCV2d Cap, and rVLVs-PCV3 Cap samples were thawed in a water bath at 42°C, and then centrifuged at 8,000 g for 5 min at 4°C to get the supernatant. Next 20 μL of sample drops were applied onto activated 200-mesh copper mesh grids and allowed to stand for 5 min. This process was repeated for 2 to 3 copper grids per sample. Apply 50 μL of 3% phosphotungstic acid onto the copper mesh, and allow it to stand for 10 min. Transfer the sample to a transmission electron microscope and observe at an operating voltage of 80 kV.

### 2.8 Studies of pathogenicity and immunogenicity

The pathogenicity of rVLVs-EGFP, rVLVs-PCV2d Cap, and rVLVs-PCV3 Cap was evaluated. Twenty-eight 6-8-week-old male C57 mice were randomly divided into four groups (*n* = 7). One group of mice was set up as a control (DMEM), and the other three groups were injected intramuscularly with 10^5^ FFU of rVLVs-EGFP, rVLVs-PCV2d cap, and rVLVs-PCV3 cap. Body weight, temperature, and survival data were recorded daily for 14 days after infection.

The immunogenicity of rVLVs-PCV2d Cap and rVLVs-PCV3 Cap was evaluated. Groups of 6-8-week-old C57 mice were immunized i.m. with a high dose (10^5^ FFU) or low dose (10^3^ FFU) of rVLVs-PCV2d Cap and rVLVs-PCV3 Cap, respectively. One group of mice was treated with DMEM as a negative control, while another group received 10^5^ FFU of the WH strain as a positive control. Sera samples from immunized mice were collected weekly until day 28 after immunization.

### 2.9 Hematoxylin and eosin staining

Fourteen 6-8-week-old C57 mice were randomly divided into two groups (*n* = 7). One group of mice was set up as a control (DMEM), and the other groups of mice were immunized i.m. with 10^5^ FFU rVLVs-EGFP, rVLVs-PCV2d Cap, and rVLVs-PCV3 Cap. At 7 days post-infection, four groups of mice were euthanized using the cervical dislocation method, and major organs (heart, liver, spleen, lung, kidney, and brain) along with duodenum were removed and fixed with 4% paraformaldehyde. The tissue and serum samples were collected at the end of the experiment. All procedures were performed per the Shanxi Agricultural University Guidelines for the Care and Use of Laboratory Animals. All the fixed organs were embedded in paraffin. The tissues were cut into 4 μm thick and stained using hematoxylin–eosin (H&E).

### 2.10 Enzyme-linked immunosorbent assay

To detect specific antibodies, a commercial ELISA kit was used to determine the titers of PCV2d-capsid-specific IgG and PCV3-capsid-specific IgG in the serum collected from each mouse, according to the manufacturer’s instructions (Rising Biotechnology Ltd., Quanzhou, China). Briefly, microtiter plates were coated with the PCV2 antigen. Mouse sera were diluted 1:50 and incubated with the antigen for 30 min at 37°C followed by five washes. Horseradish Peroxidase (HRP)-labeled goat anti-mouse IgG and then added to each well and incubated for another 30 min at 37°C. After washing, TMB substrate was added and incubated for 10 min at 37°C, protected from light, and the termination solution was added and then analyzed by an enzyme marker (Lifeorigin Technology Co. China) to determine optical density at 450 nm.

For the detection of IL-4 and IFN-γ, sera collected on day 28 post-immunization were assayed using a commercial ELISA kit (Meimian Biotechnology Co., Ltd., Yancheng, China) following the manufacturer’s instructions.

### 2.11 Statistical analysis

Statistical analysis was performed using Graph Pad Prism 8.0 software. Data distribution was assessed using the Shapiro–Wilk test, and all data passed the normality test. One-way ANOVA was used for comparisons between groups. Error bars represent the standard deviation of the mean (SD). *p* < 0.05 was considered significant.

## 3 Results

### 3.1 rVLVs construction and expression

**Figure 1:**
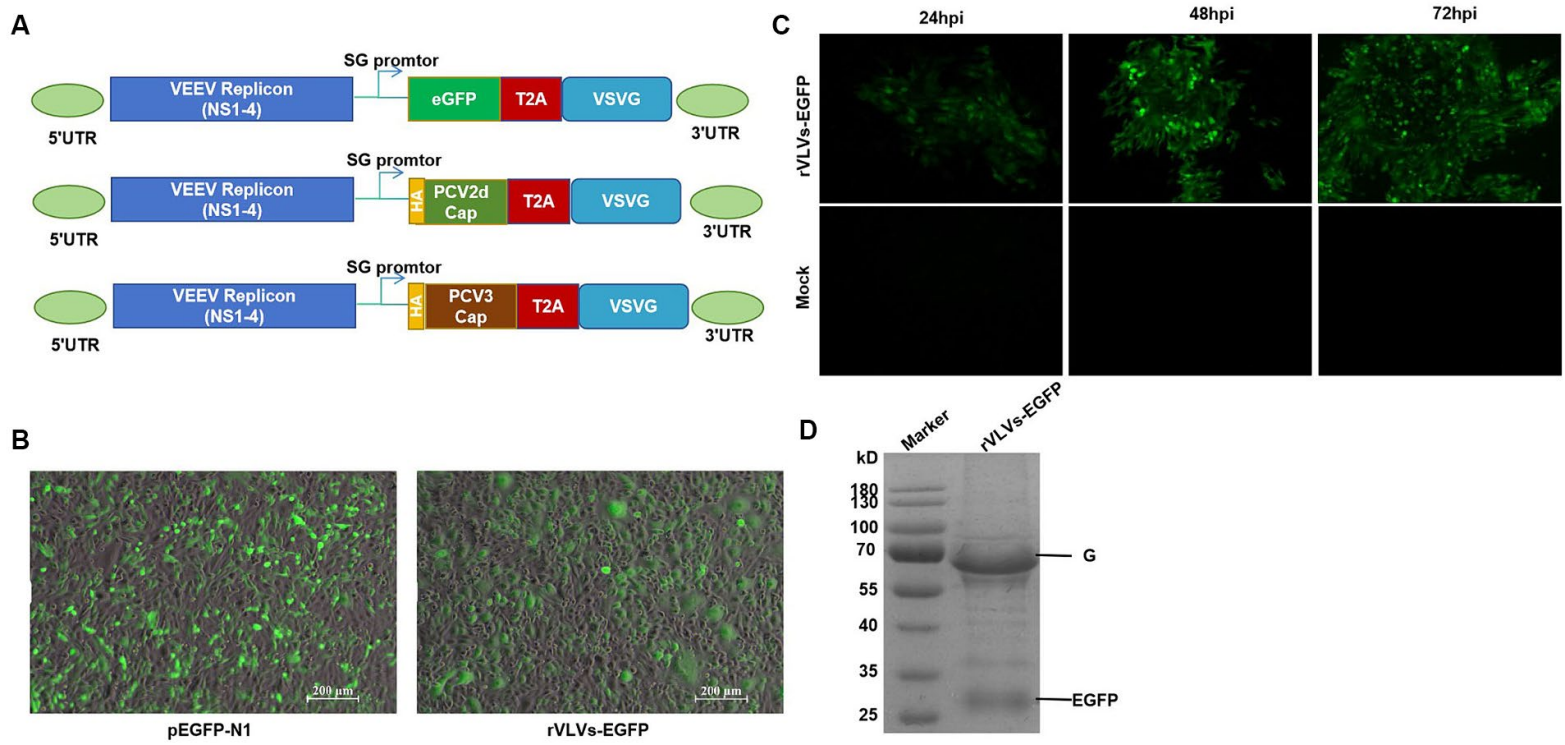
Construction and identification of rVLVs-EGFP. **(A)** Diagram of VLV DNA constructs used to generate rVLVs expressing EGFP, PCV2d Cap, and PCV3 Cap proteins. Sequences encoding the EGFP, PCV2d Cap, and PCV3 Cap proteins are inserted under the viral subgenomic RNA promoter upstream of a T2A ribosomal skipping site and the VSV G protein. **(B)** Fluorogram of BHK-21 cells transfected with rVLVs-EGFP mRNA at 72 hpt post-transfection. A transfection of the pEGFP-N1 plasmid was used as a positive control. **(C)** Fluorogram of rVLVs-EGFP infected BHK-21 cells. BHK-21 cells were infected with supernatants harvested after transfection of BHK-21 cells with rVLVs-EGFP genomic RNA at 72 hpt. At the indicated time points, the expression of the RABV-G protein was analyzed by the accumulation of green fluorescence. **(D)** SDS-PAGE analysis of supernatants from BHK-21 cells infected with rVLVs-EGFP. The rVLVs-EGFP was separated by a 12% SDS-PAGE gel, and the protein bands were observed by coomassie staining. The position and molecular weight of the rVLVs-EGFP structural protein VSV-G were also indicated.

We constructed VLV vectors expressing the EGFP protein, PCR-amplified the EGFP and VSV-G coding sequences, and a ribosomal T2A skipping site was constructed between EGFP and VSV-G. These fragments were then cloned into the α-virus VEEV replicon expression vector such that the EGFP gene was inserted into the ribosomal T2A skipping site (23) and upstream of the VSV-G protein (Figure 1A). The T2A site facilitated the expression of both EGFP and VSV-G proteins from the same subgenomic promoter, ensuring that EGFP protein expression occurred during the read-through stage before VLV production. As outlined previously, separate VLV vectors expressing PCV2d-Cap or PCV3-Cap were constructed (Figure 1A) by replacing the EGFP sequences with the PCV2d Cap and PCV3 Cap sequences.

Chimeric VEEV replicon RNA (VEEV-EGFP-VSV-G) was transfected into BHK-21 cells, and pEGFP-N1 plasmid was used as a positive control. EGFP expression was verified by observing specific green fluorescence. Visible at 72 h post-transfection (hpt), clear green fluorescence was observed, consistent with the positive control (Figure 1B). Next, we determined the infectivity of the rVLV-EGFP cells. The cell culture supernatant obtained from the transfected cells at 72 hpt, namely the P0 generation rVLVs-EGFP, was used to infect BHK-21 cells. A time-dependent accumulation of positive cells in the infected group was observed from 24 to 72 hpi, indicating the production of infectious VLVs expressing the EGFP protein (Figure 1C). The above results demonstrate that rVLV-EGFP is viable and capable of infecting BHK-21 cells.

### 3.2 *In vitro* characterization of rVLVs

**Figure 2:**
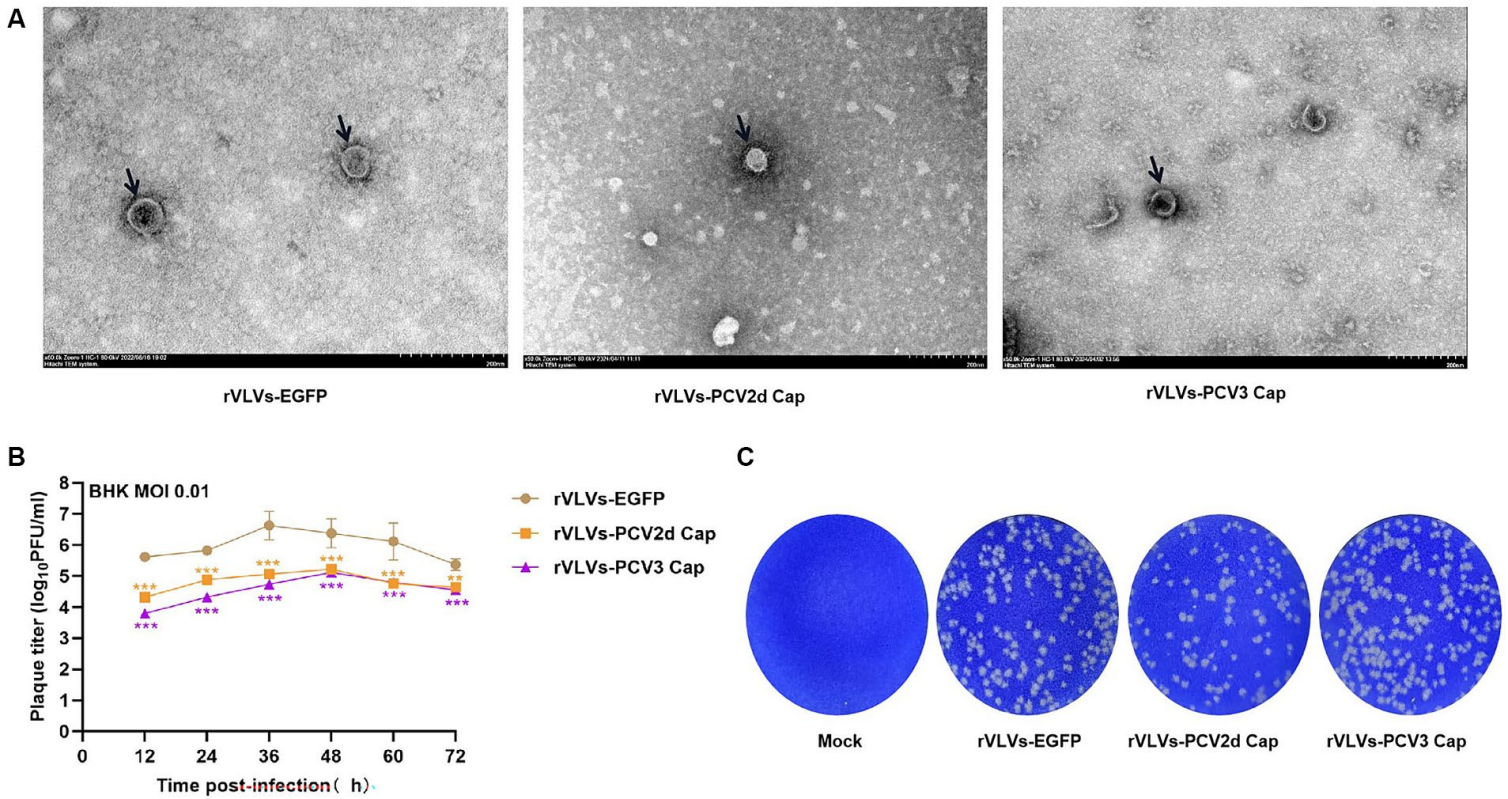
Characterization of rVLVs-EGFP, rVLVs-PCV2d Cap, and rVLVs-PCV3 Cap. **(A)** Transmission electron microscopy of rVLVs-EGFP, rVLVs-PCV2d Cap, and rVLVs-PCV3 Cap. Scale bars of TEM images are shown as indicated. **(B)** Comparison of viral growth kinetics of rVLVs-EGFP, rVLVs-PCV2d Cap, and rVLVs-PCV3 Cap viruses. The viral growth curves were plotted at an MOI of 0.01. **(C)** Plaque morphology comparison between rVLVs-EGFP, rVLVs-PCV2d Cap, and rVLVs-PCV3 Cap in BHK-21 cells. Data are presented as means ± SD. Asterisks indicate significant differences between the indicated experimental groups, ^**^p < 0.01, ^***^p < 0.001 (One-way ANOVA). Orange asterisks represent the difference between rVLVs-PCV2d Cap and rVLVs-EGFP and Purple asterisks represent the difference between rVLVs-PCV3 Cap and rVLVs-EGFP.

To study the *in vitro* characteristics of rVLVs-EGFP, rVLVs-PCV2d Cap, and rVLVs-PCV3 Cap. First, we infected a large number of BHK21 cells with rVLVs-EGFP, rVLVs-PCV2d Cap, and rVLVs-PCV3 Cap at an MOI of 0.01. The collected rVLV-EGFP cell supernatants were analyzed using SDS-PAGE (Figure 1D). As expected, cell supernatants contained EGFP and VSV-G proteins. The rVLVs-EGFP, rVLVs-PCV2d Cap, and rVLVs-PCV3 Cap vesicles were purified by sucrose density centrifugation. The morphologies of rVLVs-EGFP, rVLVs-PCV2d Cap, and rVLVs-PCV3 Cap were observed using transmission electron microscopy (TEM). In contrast to the typical 180 nm × 75 nm bullet-like structure of VSV (24), rVLVs-EGFP, rVLVs-PCV2d Cap, and rVLVs-PCV3 Cap are spherical vesicles with sizes ranging from 50 to 90 nm (Figure 2A). Overall, the results suggest that rVLVs-EGFP, rVLVs-PCV2d Cap, and rVLVs-PCV3 Cap are capable of assembling virus-like vesicles that differ significantly in shape and size from VSV.

To further investigate the characteristics of rVLVs-EGFP, rVLVs-PCV2d Cap, and rVLVs-PCV3 Cap, BHK-21 cells were infected with an MOI of 0.01. The results showed that 36 hpi rVLVs-EGFP had the highest titer of 4.3 × 10^6^ FFU/mL, and 48 hpi rVLVs-PCV2d Cap and rVLVs-PCV3 Cap had the highest titer of 1.7 × 10^5^ FFU/mL and 1.3 × 10^5^ FFU/mL, respectively (Figure 2B). Empty spots of approximately the same size and shape were also observed (Figure 2C).

### 3.3 Genetic stability of rVLVs-PCV2d cap and rVLVs-PCV3 cap

**Figure 3:**
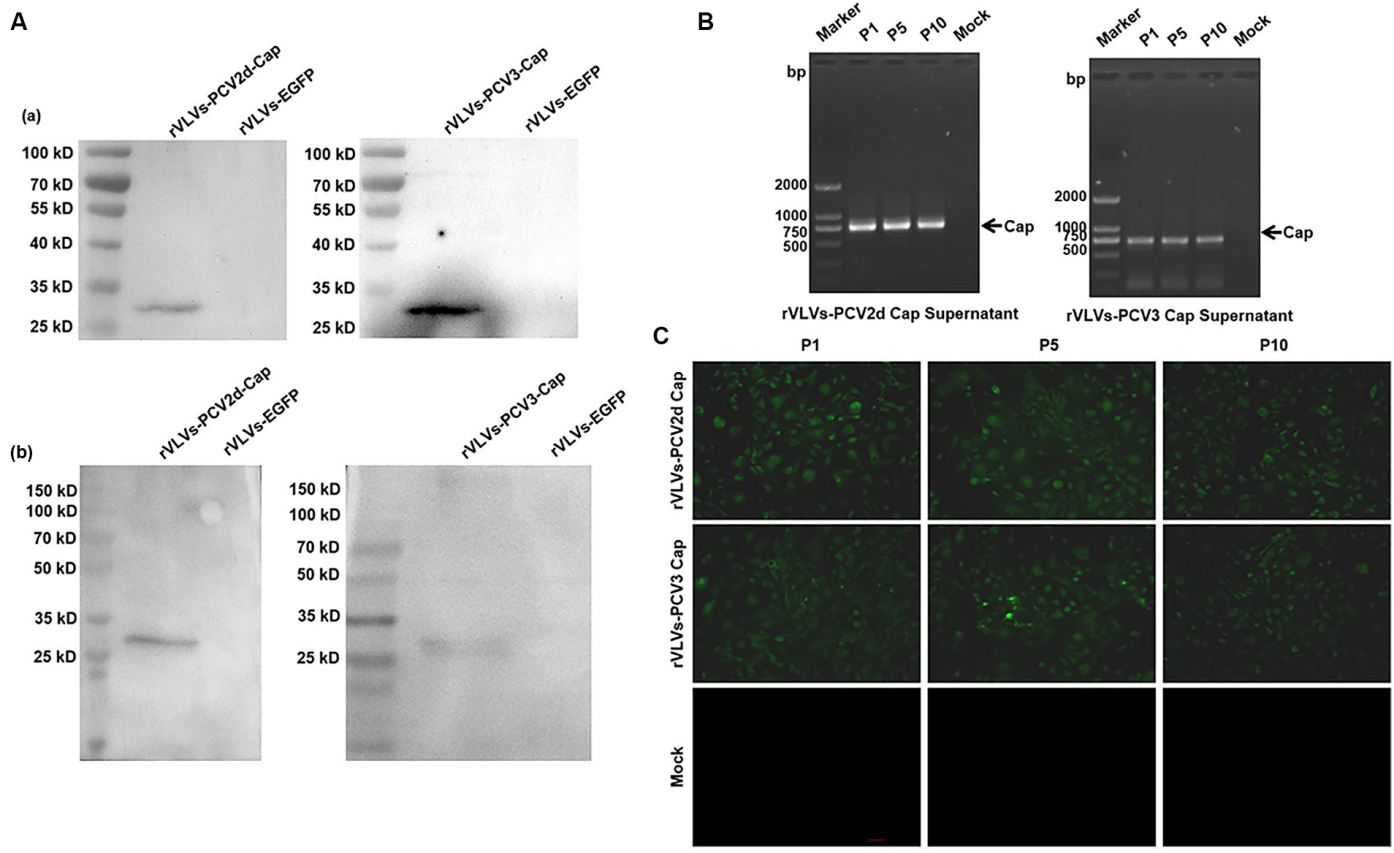
Genetic stability of rVLVs-PCV2d Cap and rVLVs-PCV3 Cap. **(A)** Western Blotting analysis of PCV2d Cap and PCV3-Cap protein expression in cell culture from rVLVs-PCV2d Cap and rVLVs-PCV3 Cap infected cells. **(a)** The anti-HA-tag mAb as the primary antibody. **(b)** The sera of infected pigs with PCV2 or PCV3 as the primary antibody. **(B)** RT-PCR analysis of viral genome stability during passage. **(C)** IFA analysis of PCV2d Cap and PCV3-Cap protein expression in P1, P5, and P10 rVLVs-PCV2d Cap and rVLVs-PCV3 Cap using HA murine monoclonal antibody.

Rescue of the rVLVs-PCV2d cap and rVLVs-PCV3 Cap on BHK-21 cells was based on the method described above. The expression of PCV2d and PCV3 Cap protein in cell lysates from rVLVs-PCV2d Cap and rVLVs-PCV3 Cap at 48 hpi was analyzed by western blotting. The PCV2d Cap and PCV3 Cap proteins produced in BHK cells were detected by SDS-PAGE (Figure 3A).

To verify the stability of rVLVs-PCV2d Cap and rVLVs-PCV3 Cap during passaging, we passaged rVLVs-PCV2d Cap and rVLVs-PCV3 Cap on BHK-21 cells for 10 passages and collected the cell culture supernatants. BHK21 cells were characterized for PCV2d-Cap and PCV3-Cap protein expression (Figure 3A). The presence of PCV2d-Cap and PCV3-Cap genes was confirmed by TR-PCR and IFA in both P1 and P10 passages of the virus-like vesicles (Figures 3B,C). The results showed that rVLVs-PCV2d Cap and rVLVs-PCV3 Cap had high genetic stability, were able to pass continuously through cell lines, and stably expressed PCV2d-Cap and PCV3-Cap proteins.

### 3.4 VLV pathogenicity in mice

**Figure 4:**
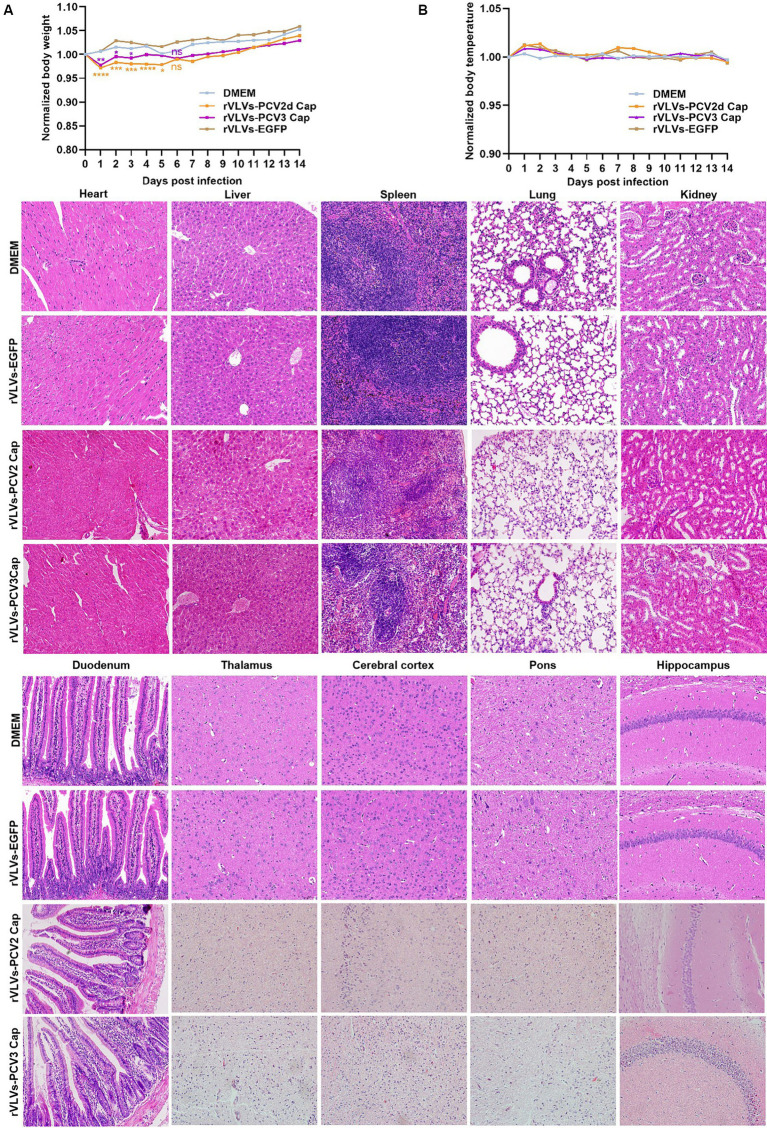
Virulence of rVLVs in mice. **(A,B)** Groups of 6-week-old male C57 mice (*n* = 7/group) were via i.m. infected with rVLVs-EGFP, rVLVs-PCV2d Cap, and rVLVs-PCV3 Cap 105 FFU or mock-infected with 100 μL DMEM. **(A)** Body weight loss was monitored daily for 14 days, and **(B)** body temperature was monitored daily for 14 days. **(C)** Groups of 6-week-old male C57 mice (*n* = 7/group) were i.m. injected with rVLVs-EGFP, rVLVs-PCV2d Cap, and rVLVs-PCV3 Cap 105FFU or mock-infected with 100 μL DMEM. At 7 d.p.i., all groups of mice were euthanized, parenchymal organs such as the heart, liver, spleen, lungs, kidneys, and brain and as well as the hollow organ duodenum were taken to make paraffin tissue sections for HE staining, and histopathological analysis. Sagittal sections of the brain were H&E stained and histopathologically analyzed in different brain regions including the pons, thalamus, hippocampus, cerebral cortex, and presented representative histological changes. Data are presented as means ± SD. Asterisks indicate significant differences between the indicated experimental groups, ^*^p < 0.05, ^**^p < 0.01, ^***^p < 0.001 (One-way ANOVA). Orange asterisks represent the difference between rVLVs-PCV2d Cap and DMEM and Purple asterisks represent the difference between rVLVs-PCV3 Cap and DMEM.

We infected 6-week-old C57 mice with 10^5^ FFU of rVLVs-EGFP, rVLVs-PCV2d Cap, and rVLVs-PCV3 Cap via i.m., and established a negative control using DMEM. Changes in body weight and temperature, and survival rates of the mice were observed and recorded up to 14 days post-infection (d.p.i). No significant differences in body weight loss were observed between the rVLV-EGFP- and control-infected mice. However, there was a significant body weight loss between the rVLVs-PCV2d Cap and rVLVs-PCV3 Cap-infected mice and mice in the DMEM group on day 1 post-infection. Subsequently, body weight gradually increased and recovered to levels of the DMEM group by 6 dpi (Figure 4A), with no observed deaths. Additionally, no differences were observed between rVLVs-EGFP, rVLVs-PCV2d Cap, and rVLVs-PCV3 Cap-infected compared to the control-infected mice (Figure 4B).

To further evaluate the safety of VLV expression of exogenous antigenic genes, we analyze organ pathological changes induced by intramuscular infection using hematoxylin and eosin (H&E) staining (Figure 4C). 6-week-old C57 mice were intramuscularly inoculated with rVLVs-EGFP, rVLVs-PCV2d Cap, rVLVs-PCV3 Cap, or DMEM. At 7 d.p.i., the parenchymal organs and duodenum of mice were removed and subsequently stained with H and E to detect pathological changes in organs; no significant pathological changes were observed in the organs of mice in the rVLVs-EGFP rVLVs-PCV2d Cap, and rVLVs-PCV3 Cap groups compared with the DMEM group. These results indicate that rVLV-infected mice do not exhibit pathological changes and demonstrate the safety of the approach.

### 3.5 rVLVs-PCV2d cap and rVLVs-PCV3 cap immunogenicity in mice

**Figure 5:**
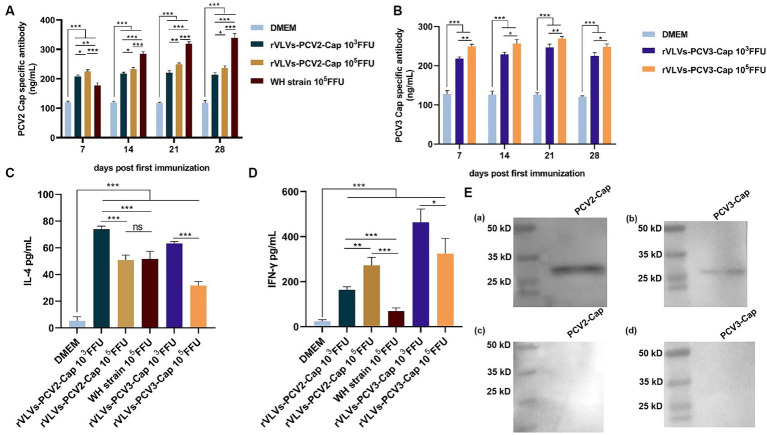
Humoral and cellular immunity of rVLVs-PCV2d Cap and rVLVs-PCV3 Cap immunized C57 mice. **(A)** ELISA to detect the titer of PCV2b-capsid-specific IgG in the serum of immunized mice. **(B)** ELISA to detect the titer of PCV3-capsid-specific IgG in the serum of immunized mice. Serum levels of **(C)** IFN-γ and **(D)** IL-4 cytokines in 28 d.p.i. mice were determined by ELISA. Data were expressed as mean ± SD, and comparisons between groups were made by One-way ANOVA, ^*^p < 0.05, ^**^p < 0.01, ^***^p < 0.001. **(E)** Western Blotting was performed to verify the recognition of PCV2 and PCV3 Cap by the sera of immunized mice with rVLVs-PCV2 Cap and rVLVs-PCV3 Cap. **(a,b)** The sera of immunized mice with rVLVs-PCV2 Cap and rVLVs-PCV3 Capas primary antibody. **(c,d)** The sera of immunized mice with DMEM as primary antibody.

To assess the ability of rVLVs-PCV2d Cap and rVLVs-PCV3 Cap to stimulate humoral immunity in mice, we measured antibody levels in the serum of mice every 7 days. The results revealed that mice immunized with WH strain, rVLVs-PCV2d Cap, and rVLVs-PCV3 Cap exhibited significantly higher Cap-specific antibody titers compared to mice in the DMEM group (Figures 5A,B). rVLVs-PCV2d Cap and rVLVs-PCV3 Cap immunized mice showed a similar trend in IgG antibody titers, which gradually increased from the time of immunization, peaked 21 days after immunization, and decreased 28 days after immunization. Additionally, a dose-dependent increase in cap-specific antibody titers was observed in immunized mice, with specific antibodies being significantly higher in the high-dose (10^5^ FFU) group than in the low-dose (10^3^ FFU) group. However, the antibody titer was notably higher in the WH strain group than in the rVLVs-PCV2d Cap groups (Figures 5A,B).

To assess the cytokine response to immunization with rVLVs-PCV2d Cap and rVLVs-PCV3 Cap, the serum levels of IFN-γ and IL-4 in 28 dpi mice were evaluated. Serum levels of IFN-γ and IL-4 were significantly increased in mice in either the high-dose (10^5^ FFU) group or the low-dose (10^3^ FFU) group compared with the DMEM group. The level of IFN-γ production in mice from both the rVLVs-PCV2d Cap high-dose group and the rVLVs-PCV2d Cap low-dose group was significantly higher than that in mice in the WH strain group (Figure 5D) Additionally, the level of IL-4 production in mice from the rVLVs-PCV2d Cap low-dose group was significantly lower than that in mice in the rVLVs-PCV2d Cap high-dose group and the WH strain group (Figure 5C). Moreover, mice in the rVLVs-PCV3 Cap low-dose group exhibited significantly higher levels of both IFN-γ and IL-4 production compared tp rVLVs-PCV3 Cap mice in the high-dose group (Figure 5D). These results indicated that immunization with rVLVs-PCV2d Cap and rVLVs-PCV3 Cap could enhance the Th1/Th2 immune response in mice. Overall, immunization with rVLVs-PCV2d Cap and rVLVs-PCV3 Cap at a low dose (10^3^ FFU) resulted in significantly higher cytokine levels compared to immunization with a high dose (10^5^ FFU) and the WH strain.

## 4 Discussion

Alphaviruses are positive-stranded RNA viruses, including the Sindbis virus (SINV), Simenic Forest virus (SFV), and Venezuelan equine encephalitis virus (VEEV). Similar to the SFV and SINV replication subsystems, VEEV vectors are capable of the high-level synthesis of heterologous proteins in a wide range of cell types from different species. Recently, it has been shown that in addition to the SFV replicon expression of VSV-G or RABV-G (19, 25), infectious VLVs can be obtained by utilizing the VEEV replicon expression of RABV-G (26). This suggests that infectious VLVs can be formed by expressing VSV-G using different metaviral replicons. In this study, we first modified rVLVs with VEEV replicons expressing VSV-G using a reverse genetic manipulation technique, introduced the EGFP gene, and explored their expression in BHK-21 cells and their pathogenicity in mice. Preliminary validation of its *in vitro* characterization was conducted through a series of experiments. The successful rescue of rVLV-EGFP, which stably inherits and expresses exogenous proteins, demonstrates the feasibility of using VEEV-VSV-G as a vector to express exogenous proteins through the T2A sequence. Insertion of the EGFP gene and 2A sequence did not affect the structure and proliferation of rVLVs, as determined by electron microscopy and other experiments.

Based on the above studies, we inserted the PCV2d Cap and PCV3 Cap genes into the VEEV-VSV-G vector, constructed rVLVs-PCV2d Cap and rVLVs-PCV3 Cap expressing PCV2d Cap and PCV3 Cap proteins, respectively, and used indirect immunofluorescence assays and western blotting to confirm the successful rescue of rVLVs. Stable inheritance and expression of PCV2d Cap and PCV3 Cap were confirmed in BHK-21 cells. rVLVs-PCV2d Cap and rVLVs-PCV3 Cap maintained stable inheritance and expression of the PCV2d Cap and PCV3 Cap genes after 10 consecutive passages. In addition, the growth characterization of rVLVs showed that the viral titer of rVLV-eGFP was highest at 36 hpi, whereas the viral titers of rVLVs-PCV2d Cap and rVLVs-PCV3 Cap were highest at 48 hpi and were significantly lower than those of rVLV-eGFP. rVLVs-PCV2d Cap and rVLVs -PCV3 Cap had significantly different growth characteristics than rVLV-eGFP. Thus, at the cellular level, the expression of PCV2d Cap and PCV3 Cap proteins affected the proliferation of rVLVs.

A significant advantage of rVLVs as vaccines is their high safety profile. Compared to other recombinant viral vector vaccines (27–30), rVLVs have demonstrated an excellent safety profile in mouse models. Our data showed that all adult mice showed no weight loss, with a survival rate of 100% and no significant histopathological damage. *In vivo*, studies demonstrated that the immunization of mice with rVLVs-PCV2d Cap and rVLVs-PCV3 Cap efficiently induced the production of high titers of PCV2 and PCV3 capsule-specific antibodies. Furthermore, rVLVs-PCV2d Cap immunization produced significantly lower antibody levels than the WH strain. IFN-γ is an important Th1-type cytokine with an important role in the cellular immune response, and IL-4 is an important Th2-type cytokine with an important role in the humoral immune response. Immunization with rVLVs-PCV2d Cap and rVLVs-PCV3 Cap significantly enhanced the production of IFN-γ and IL-4. Interestingly, the low-dose (10^3^ FFU) group induced significantly higher levels of IFN-γ and IL-4 than the high-dose (10^5^ FFU) group and the WH strain group. In summary, the rVLVs-PCV2d Cap and rVLVs-PCV3 Cap developed in this study efficiently triggered cellular and humoral immune responses in mice at a low dose (10^3^ FFU).

In summary, rVLVs-PCV2d Cap and rVLVs-PCV3 Cap were able to spontaneously formed virus-like vesicles. These virus-like vesicles not only induced the production of specific capsid antibodies but also enhanced the Th1/Th2 immune response, suggesting that rVLVs-PCV2d Cap and rVLVs-PCV3 Cap induced both humoral and cellular immune responses in the body. In conclusion, our results suggest that rVLVs have the potential to be developed and may be a generalized strategy for exploring novel vaccines against other viruses.

## Data availability statement

The original contributions presented in the study are included in the article/Supplementary material, further inquiries can be directed to the corresponding authors.

## Ethics statement

The animal study was approved by the Institutional Animal Care and Use Committee of Experimental Animal Management at the Shanxi Agricultural University, China. The study was conducted in accordance with the local legislation and institutional requirements.

## Author contributions

YW: Writing – review & editing, Writing – original draft. MS: Writing – review & editing, Writing – original draft. YH: Writing – review & editing. JR: Writing – review & editing. SN: Writing – review & editing. YZ: Writing – review & editing. FY: Writing – review & editing. YY: Writing – review & editing. W-xT: Writing – review & editing.
