# Supplementary material for: Development of a novel PCV2 and PCV3 vaccine using virus-like vesicles incorporating Venezuelan equine encephalomyelitis virus-containing vesicular stomatitis virus glycoprotein
Source: Front Vet Sci. 2024 May 22;11:1359421. doi: 10.3389/fvets.2024.1359421 (PMC11150706; doi:10.3389/fvets.2024.1359421)
Supplement: Supplementary file 1 [file Table_1.DOCX]

## Supplementary Table S1. Primers used in this study.

| **Names of primer** | **Sequence (5′-3′)** |
| --- | --- |
| EGFP-F | GTCTAGTCCGCCAAGTCTAGGCCACCATGGTGAGCAAGGGCGAGGAGCTGTTC |
| EGFP-2A-R | CGCATGTTAGCAGACTTCCTCTGCCCTCCTTGTACAGCTCGTCCATGCCGAGAGTGATC |
| 2A-VSV G-F | TCGAGGAGAATCCTGGCCCAATGAAGTGCCTTTTGTACTTAGCCTTTTTATTCATTGGG |
| VSV G-R | ACGCGTCGAGGGGAATTAATTCTTGAAGACAATAAGTAATGTAAAATACAGCATAGCC |
| PCV2d Cap-F | ATGTACCCATACGATGTTCCAGATTACGCTACGTATCCAAGGAGGCGTTTCCGCAGACG |
| PCV2d Cap-R | GTTAGCAGACTTCCTCTGCCCTCCTTAGGGTTAAGTGGGGGGTCTTTAAGATTAAATTC |
| PCV3 Cap-F | ATGTACCCATACGATGTTCCAGATTACGCTAGACACAGAGCTATATTCAGAAGAAGACC |
| PCV3 Cap-R | GTTAGCAGACTTCCTCTGCCCTCGAGAACGGACTTGTAACGAATCCAAACTTCTTTG |
| HA-F | ACATAGTCTAGTCCGCCAAGTCTAGGCCACCATGTACCCATACGATGTTCCAGATTACG |
